# Outcomes of concurrent chemoradiotherapy versus chemotherapy alone for stage IV esophageal squamous cell carcinoma: a retrospective controlled study

**DOI:** 10.1186/s13014-018-1183-y

**Published:** 2018-11-26

**Authors:** Jiahua Lyu, Tao Li, Qifeng Wang, Fang Li, Peng Diao, Li Liu, Churong Li, Jinyi Lang

**Affiliations:** Department of Radiation Oncology, Sichuan Cancer hospital institute, Sichuan Cancer Center, School of Medicine, University of Electronic Science and Technology of China, No.55, 4th section of Renmin South Road Chengdu, Chengdu, 610041 Sichuan Province People’s Republic of China

**Keywords:** Chemoradiotherapy, Esophageal squamous cell carcinoma, Chemotherapy, Primary tumor response rate, Overall survival, Progression-free survival

## Abstract

**Background:**

The purpose of this study is to compare the efficacy and safety of concurrent chemoradiotherapy (CCRT) versus chemotherapy alone for patients with stage IV esophageal squamous cell carcinoma (ESCC).

**Methods:**

Eligible patients were retrospectively enrolled at the authors’s institution from January 2010 to October 2015. Of the 141 patients enrolled, 55 (39.0%) received CCRT and 86 (61.0%) received chemotherapy alone. The outcomes and adverse events (AEs) were compared between the two groups.

**Results:**

The baseline clinical characteristics of the two groups were similar. However, the CCRT group showed a significantly better primary tumor objective response rate (ORR) than that of the chemotherapy group (74.5% versus 45.3%, *p* = 0.001). The 1-year, 2-year, 3-year overall survival (OS) rates and median OS were 58.0% versus 43.0%, 25.5% versus 14.0%, 10.7% versus 4.7%, and 14 months versus 11 months for patients treated with CCRT or chemotherapy, respectively (*p* = 0.007). The 1-year and median progression-free survival (PFS) were 29.8% versus 14.9% and 8 months versus 6 months (*p* = 0.005). Multivariate analysis identified CCRT (*p* = 0.013) and solitary metastasis (*p* = 0.037) as independent factors for greater OS. The frequency of leucocytopenia (grade 3 or higher) was significantly higher in the CCRT group than in the chemotherapy-alone group (*p* = 0.040), whereas the rates of other AEs did not differ.

**Conclusions:**

In this study, it is suggested that CCRT is more effective than chemotherapy alone for stage IV ESCC, yielding better primary responses and survival outcomes with tolerable side effects.

## Background

Esophageal cancer (EC) is one of the most common malignant diseases. In 2012, about 455,800 new cases and 400,200 deaths were reported worldwide [1]. In China, EC is the fourth most common cause of cancer-related deaths in men and the sixth most common cause in women [2]. There are two main histological types of EC: squamous cell carcinoma (SCC) and adenocarcinoma. Esophageal squamous cell carcinoma (ESCC) is more common in Asian countries and accounts for more than 90% of EC cases in China.

Approximately 18–40% of patients with EC present with distant metastasis to the lungs, liver, bone, or nonregional lymph nodes at diagnosis [3–5]. The current National Comprehensive Cancer Network (NCCN) guidelines recommend chemotherapy only and/or palliative/best supportive care for patients with metastatic EC [6]. Regardless of the therapeutic strategy, however, metastatic EC carries a poor prognosis, with a reported median survival of only six to eight months and five-year survival rates of less than 5% [7–9].

Radiotherapy is generally not used as first-line therapy for stage IV EC, although it can be used as supportive and palliative care for controlling esophageal bleeding and relieving obstruction and dysphagia caused by esophageal primary tumors. Concurrent chemoradiotherapy (CCRT) is the standard treatment for patients with inoperable locally advanced EC, but little information is available on the potential benefits of CCRT for stage IV ESCC. Further, the toxicity of such therapy is unclear. We, therefore, retrospectively compared outcomes, including primary tumor response rates, overall survival (OS), progression-free survival (PFS), and safety profiles, among patients with stage IV ESCC, who were treated with CCRT or chemotherapy alone.

## Methods

### Patient selection criteria

We retrospectively reviewed all patients with stage IV ESCC who were treated at the author’s institution from January 2010 to October 2015. Staging was determined according to the 7th (2009) edition of the American Joint Committee on Cancer staging system [10]. The inclusion criteria were as follows: histologically confirmed SCC of the esophagus; age 18–75 years; Karnofsky Performance Status ≥70; clinical stage TanyNanyM1 with nonregional lymph node or distant organ metastasis; treatment by a combination of cisplatin (CDDP) plus 5-fluorouracil (5-FU) or paclitaxel (PTX) chemotherapy with or without concurrent radiotherapy to the primary tumor; sufficient pretreatment assessment; and sufficient follow-up data available for tumor response, toxicity, and survival assessment. The exclusion criteria were age < 18 or > 75 years, insufficient follow-up data, incomplete chemotherapy (patients received chemotherapy but failed to complete the whole course of one chemotherapy cycle) or CCRT (radiotherapy dose <50Gy), previous malignancy or other concomitant malignant diseases, and treatment with molecular-targeted therapeutic drugs.

### Treatment

The chemotherapeutic regimen comprised CDDP combined with 5-FU or PTX in all patients. Cisplatin (25 mg/m^2^/day) was administered intravenously on days 1–3, and 5-FU (500 mg/m^2^/day) was administered by continuous infusion for 24 h on days 1–5. PTX (135 mg/m^2^) was administered intravenously over 3 h on day 1. Chemotherapy was performed and repeated every three weeks.

Among the included patients, 55 (39.0%) patients underwent concurrent radiotherapy for controlling esophageal bleeding or relieving dysphagia. Intensity-modulated radiation therapy (IMRT) was delivered at a median dose of 56.4 Gy (range: 50.0–66.0 Gy), generally in once-daily 1.8–2.0 Gy fractions. IMRT treatment plans were generated using the Pinnacle planning system, with beam arrangement optimized for each patient. The irradiation area included the primary esophageal tumor and positive mediastinal lymph nodes if present. Metastatic lesions were not routinely included in the radiation area unless serious symptoms caused by metastatic lesions were observed.

### Assessment of primary tumor responses, survival, and toxicity

The primary tumor response was assessed according to the Response Evaluation Criteria in Solid Tumors version 1.1 as follows: complete response (CR), partial response (PR), stable disease (SD), and progressive disease (PD). The objective response rate (ORR) included CR and PR. The disease control rate (DCR) included CR, PR, and SD. OS was measured from the first day of treatment to the day of mortality or last follow-up. PFS was defined as the time between the initiation of treatment and disease progression or mortality from any cause. Treatment-associated adverse events (AEs) were graded by the National Cancer Institute’s Common Terminology Criteria for Adverse Events (version 4.0) and the Radiation Therapy Oncology Group criteria [11, 12].

### Statistical analysis

Data were collected retrospectively. Student’s *t*-tests were used to compare differences in continuous variables and chi-square tests were used to compare categorical variables between CCRT and chemotherapy-alone groups. PFS and OS were estimated using the Kaplan–Meier method and compared between groups using the log-rank test. Univariable and multivariable Cox proportional hazards regression analyses were conducted using the Enter selection method in order to test the associations between OS and potential predictive factors. All variables with a *p*-value of less than 0.20 by univariable analysis were entered into the multivariable model. The results are reported as hazard ratios (HRs) and 95% confidence intervals (CIs). A two-tailed *p*-value less than 0.05 was considered statistically significant. SPSS ver. 22.0 (IBM Corp., Armonk, NY, USA) was used for statistical analyses.

## Results

### Patient characteristics

Between January 2010 and October 2015, 198 patients with stage IV ESCC were treated at the author’s institution. Of these, 57 patients were excluded from the study for any one of the following reasons: receiving supportive care only (*n* = 26), not completing chemotherapy or CCRT as required (*n* = 15), insufficient follow-up data (*n* = 9), treatment with molecular-targeted therapeutic drugs (*n* = 5), or previous malignancy or other concomitant malignant diseases (*n* = 2). Data from the remaining 141 patients were collected for analysis, of whom 55 patients (39.0%) received CCRT (CCRT group) and 86 (61.0%) received chemotherapy alone (chemotherapy-alone group). Table 1 summarizes the baseline clinical characteristics of the 141 enrolled patients. There were no significant differences in patient-related and disease-related characteristics between the two treatment groups at baseline.

**Table 1:** Baseline characteristics for patients in CCRT group vs. chemotherapy group

Characteristics	CCRT (*n* = 55)	Chemotherapy (*n* = 86)	*P*
Sex			0.689
Male	43 (78.2%)	64(74.4%)	
Female	12 (21.8%)	22 (25.6%)	
Age, years			0.862
≤ 60	32 (58.2%)	48 (55.8%)	
> 60	23 (41.8%)	38 (44.2%)	
Tumor location			0.674
cervical	3 (5.5%)	3 (3.5%)	
upper thoracic	16 (29.1%)	19 (22.1%)	
middle thoracic	23 (41.8%)	38 (44.2%)	
lower thoracic	13 (23.6%)	26 (30.2%)	
KPS score			0.467
≥ 80	45 (81.8%)	64(74.4%)	
< 80	10 (18.2%)	22 (25.6%)	
T-stage			0.816
T1–2	8 (14.5%)	15 (17.4%)	
T3–4	47 (85.5%)	71 (82.6%)	
N-stage			0.797
N0	6 (10.9%)	11 (12.8%)	
N+	49 (89.1%)	75 (87.2%)	
Dysphagia score			0.096
0 (asymptomatic)	4 (7.3%)	5 (5.8%)	
1 (eat solid diet with some dysphagia)	5 (9.1%)	23 (26.7%)	
2 (eat semisolid diet)	28 (50.9%)	36 (41.9%)	
3 (drink liquid diet)	13 (23.6%)	19 (22.1%)	
4 (complete dysphagia)	5 (9.1%)	3 (3.5%)	
Number of metastatic organs			0.351
Solitary metastasis	41 (74.5%)	57 (66.3%)	
Multiple metastasis	14 (25.5%)	29 (33.7%)	
Metastasis sites			
Nonregional lymph nodes	14 (25.5%)	24 (27.9%)	0.831
lung	14 (25.5%)	16 (18.6%)	
liver	17 (30.9%)	34 (39.5%)	
bone	21 (38.2%)	36 (41.9%)	
others	5 (9.1%)	8 (9.3%)	
Chemotherapy regimens			
PTX + DDP	24 (43.6%)	42 (48.8%)	0.605
5-Fu + DDP	31 (56.4%)	44 (51.2%)	
Chemotherapy cycles			0.585
> 2	20 (36.4%)	27 (31.4%)	
≤ 2	35 (63.6%)	59 (68.6%)	

Abbreviations: *CCRT* concurrent chemoradiotherapy, *KPS* Karnofsky Performance Status, *CDDP* cisplatin, *5-FU* 5-fluorouracil, *PTX* paclitaxel

### Responses of the primary tumor

The responses of the primary tumor are summarized in Table 2. In the CCRT group, seven patients (12.7%) achieved CR and 34 (61.8%) attained PR, for a total response rate of 74.5%. In the chemotherapy-alone group, only two patients (2.3%) achieved CR and 37 (43.0%) exhibited PR. The CCRT group demonstrated significant improvements in ORR and DCR compared to the chemotherapy-alone group (ORR: 74.5% versus 45.3%, *p* = 0.001; DCR: 94.5% versus 80.2%, *p* = 0.024).

**Table 2:** Primary tumor response for patients in CCRT group vs. chemotherapy alone group

Primary tumor response	CCRT group (n = 55)	Chemotherapy group (n = 86)	*P*
CR	7 (12.7%)	2 (2.3%)	
PR	34 (61.8%)	37 (43.0%)	
SD	11 (20.0%)	30 (34.9%)	
PD	3 (5.5%)	17 (19.8%)	
ORR (CR + PR)	41 (74.5%)	39 (45.3%)	0.001
DCR (CR + PR + SD)	52 (94.5%)	69 (80.2%)	0.024

Abbreviations: *CCRT* concurrent chemoradiotherapy, *CR* complete response, *PR* partial response, *SD* stable disease, *PD* progressive disease, *ORR* objective response rate, *DCR* disease control rate

### Survival analysis

**Fig. 1:**
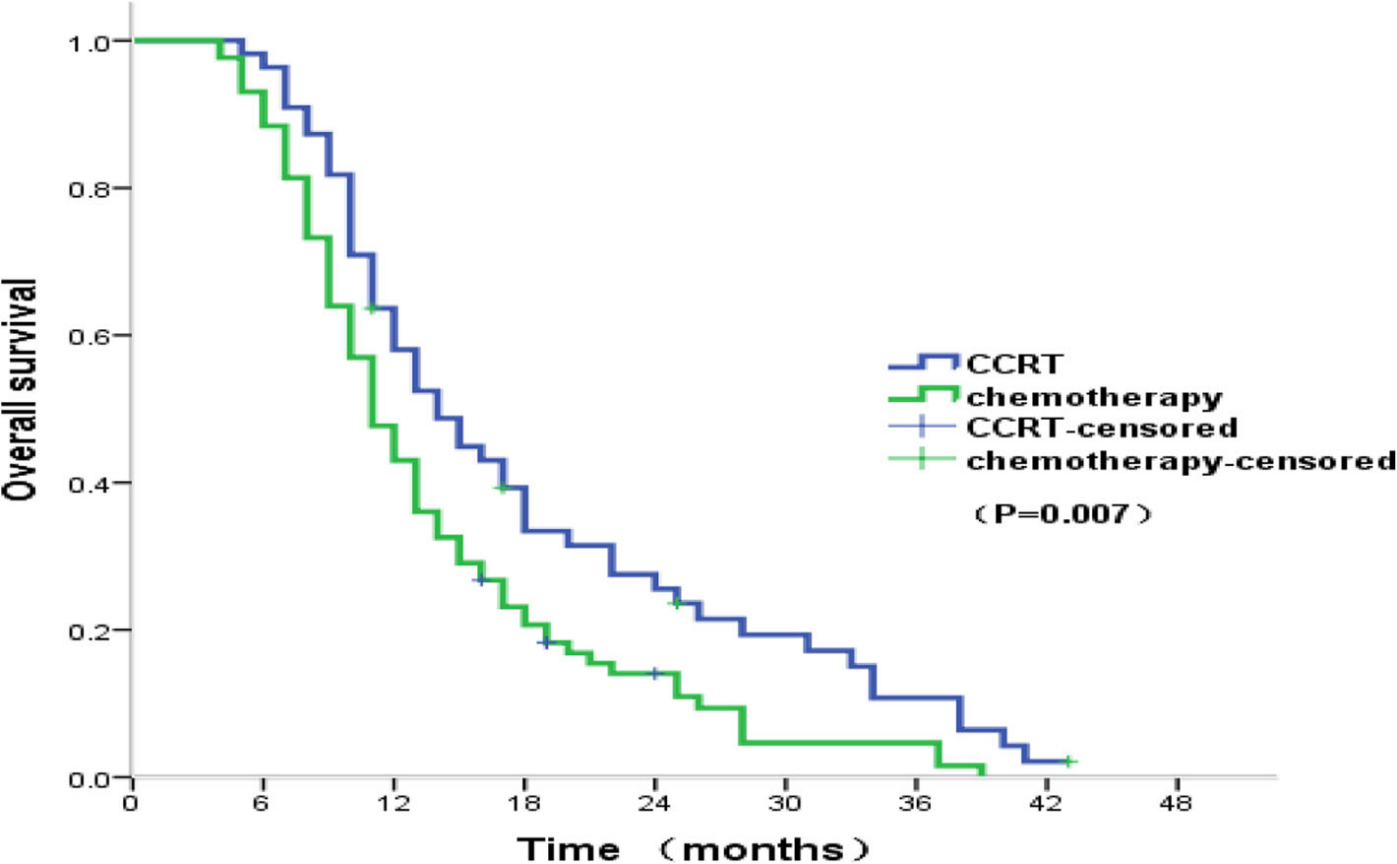
Kaplan-Meier estimates of overall survival in the CCRT and chemotherapy groups. Abbreviations: CCRT concurrent chemoradiotherapy

**Fig. 2:**
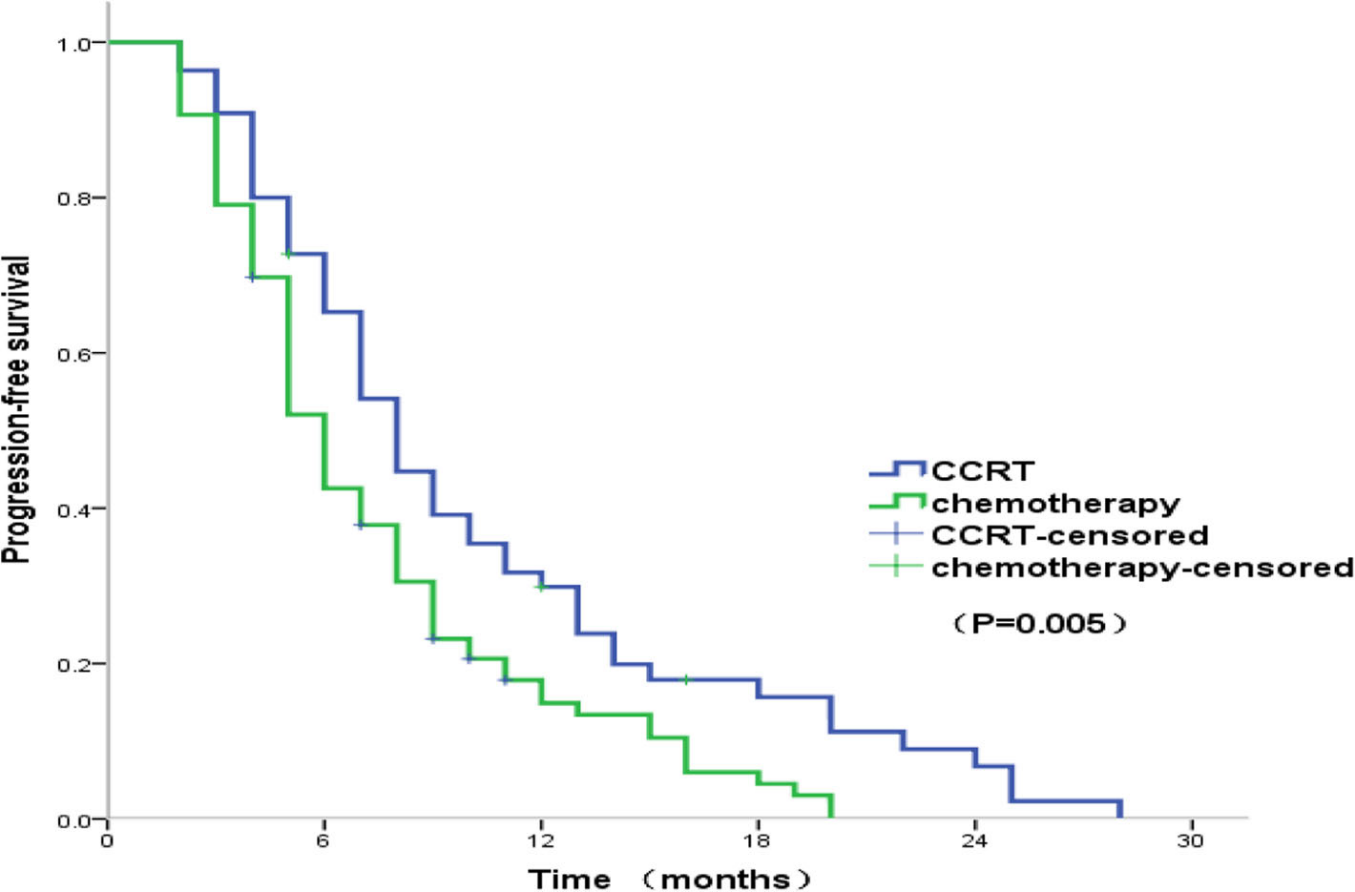
Kaplan-Meier estimates of progression-free survival in the CCRT and chemotherapy groups. Abbreviations: CCRT concurrent chemoradiotherapy

The OS rates and median OS were also higher in the CCRT group compared to the chemotherapy-alone group (one-year: 58.0% versus 43.0%; two-year: 25.5% versus 14.0%; three-year: 10.7% versus 4.7%; median: 14 months versus 11 months, *p* = 0.007) (Fig. 1). Moreover, the one-year and median PFS were also significantly higher in the CCRT group (one-year: 29.8% versus 14.9%; median: eight months versus six months, *p* = 0.005) (Fig. 2).

As summarized in Table 3, univariable analysis identified CCRT (HR: 0.626, 95% CI: 0.437–0.898, *p* = 0.011) and solitary metastasis (HR: 0.621, 95% CI: 0.426–0.965, *p* = 0.013) as potential prognostic factors for OS. Variables with a *p*-value less than 0.20 on univariable analysis, namely, treatment modality, number of metastatic organs, T-stage, and chemotherapy regimens were entered into multivariable analysis. The multivariable Cox model identified CCRT (HR: 0.631, 95% CI: 0.438-0.907, *p* = 0.013) and solitary metastasis (HR: 0.668, 95% CI: 0.457-0.976, *p* = 0.037) (Table 4) as independent factors influencing OS.

**Table 3:** Univariate analysis demonstrating factors associated with OS

Factor	OS P-value	HR (95% CI)
Treatment modality (CCRT vs chemotherapy)	0.011	0.626 (0.437–0.898)
Sex (male vs female)	0.677	0.918 (0.615–1.371)
Age (≤60 years vs > 60 years)	0.836	1.037 (0.734–1.465)
KPS score (≤80 vs > 80)	0.920	1.021 (0.679–1.536)
Primary tumor location		
cervical	reference	
upper thoracic	0.757	0.861 (0.333–2.225)
middle thoracic	0.800	1.125 (0.450–2.812)
lower thoracic	0.859	1.089 (0.427–2.774)
T- Stage (T1 + 2 vs T3 + 4)	0.096	0.667 (0.413–1.075)
N- Stage (N0 vs N+)	0.867	0.956 (0.566–1.615)
Dysphagia score		
0 (asymptomatic)	reference	
1 (eat solid diet with some dysphagia)	0.564	0.743 (0.272–2.034)
2 (eat semisolid diet)	0.615	1.239 (0.537–2.858)
3 (drink liquid diet)	0.880	1.063 (0.481–2.346)
4 (complete dysphagia)	0.870	1.072 (0.466–2.467)
Number of metastases organs (solitary vs multiple metastasis)	0.013	0.621 (0.426–0.905)
Chemotherapy regimens (PTX + DDP vs 5-Fu + DDP)	0.109	0.747 (0.523–1.067)
Chemotherapy cycles (≤2 vs > 2)	0.910	1.021 (0.711–1.466)

Abbreviations: *CCRT* concurrent chemoradiotherapy, *KPS* Karnofsky Performance Status, *CDDP* cisplatin, *5-FU* 5-fluorouracil, *PTX* paclitaxel, *HR* hazard ratio, *CI* confidence interval, *OS* overall survival

**Table 4:** Multivariate Cox regression analyses for OS

Factor	*P*	HR (95% CI)
Treatment modality (CCRT vs chemotherapy)	0.013	0.631 (0.438–0.907)
Number of metastases organs (Solitary vs Multiple metastasis)	0.037	0.668 (0.457–0.976)
T-Stage (T1 + 2 vs T3 + 4)	0.113	0.679 (0.421–1.096)
Chemotherapy regimens (PTX + DDP vs 5-Fu + DDP)	0.089	0.732 (0.511–1.049)

Abbreviations: *CCRT* concurrent chemoradiotherapy, *HR* hazard ratio, *CI* confidence interval, *OS* overall survival

### Treatment-related AEs

The hematological and nonhematological toxicities in the two groups are summarized in Table 5. Patients who received CCRT suffered more frequent grade 3 or higher leucocytopenia than those who received chemotherapy alone (41.8% versus 24.4%, *p* = 0.040). In contrast, the frequencies of thrombocytopenia, anemia, and nausea/vomiting did not differ significantly between groups (all *p* > 0.05). Eight patients in the CCRT group had grade 3 or higher treatment-related esophagitis and four patients had grade 3 or higher treatment-related pneumonitis. Treatment was well tolerated in both groups, and there were no treatment-related deaths in our study.

**Table 5:** Toxicities associated with treatment

toxicities	CCRT(N = 55)					chemotherapy(N = 86)					
	Gr.1	Gr.2	Gr.3	Gr.4	≥Gr. 3	Gr.1	Gr.2	Gr.3	Gr.4	≥Gr. 3	*P*
Leucocytopenia	11 (20.0%)	17 (30.9%)	19 (34.5%)	4 (7.3%)	41.8%	19 (22.1%)	43 (50.0%)	16 (18.6%)	5 (5.8%)	24.4%	0.040
Thrombocytopaenia	27 (49.1%)	12 (21.8%)	6 (10.9%)	0 (0.0%)	10.9%	45 (52.3%)	24 (27.9%)	7 (8.1%)	1 (1.2%)	9.3%	0.779
Anemia	37 (67.3%)	9 (16.4%)	1 (1.8%)	0 (0.0%)	1.8%	52 (60.5%)	15 (17.4%)	1 (1.2%)	0 (0.0%)	1.2%	0.647
Nausea/vomiting	28 (50.9%)	16 (29.1%)	2 (3.6%)	0 (0.0%)	3.6%	47 (54.7%)	16 (18.6%)	6 (7.0%)	0 (0.0%)	7.0%	0.483
liver injury	6 (10.9%)	1 (1.8%)	0 (0.0%)	0 (0.0%)	0%	10 (11.6%)	2 (2.3%)	0 (0.0%)	0 (0.0%)	0%	–
treatment-related pneumonitis	19 (34.5%)	7 (12.7%)	4 (7.3%)	0 (0.0%)	7.3%	–	–	–	–		–
treatment-related esophagitis	24 (43.6%)	16 (29.1%)	8 (14.5%)	0 (0.0%)	14.5%	–	–	–	–		–

Abbreviations: *CCRT* concurrent chemoradiotherapy, Gr grade

## Discussion

Patients with stage IV ESCC but in a good general condition as indicated by ECOG performance score ≤ 2 and good organ function (bone marrow, hepatic, and renal) are considered candidates for palliative chemotherapy according to current NCCN guidelines. The combination of CDDP and 5-FU is one of the most widely used chemotherapeutic regimens for advanced ESCC, but the response rate is only about 30–40% and the median survival is six to eight months [7, 9].

In recent years, PTX combined with platinum has become another widely accepted chemotherapeutic regimen for patients with locally advanced and metastatic EC. A phase II study [13] on 48 patients with advanced EC receiving 175 mg/m^2^ PTX and 80 mg/m^2^ nedaplatin until disease progression, unacceptable toxicity, or patient refusal reported an ORR of 41.7% and estimated OS values at one and two years of 43.8 and 10.4%, respectively. In addition, some new chemotherapeutic drugs, including capecitabine, docetaxel, and S-1 (DGS), have also been examined for efficacy against ESCC. A phase II study assessing the efficacy and safety of capecitabine/CDDP as first-line chemotherapy for stage IV ESCC reported an ORR of 57.8%, median PFS of 4.7 months, and OS of 11.2 months [14]. In another phase II study on 43 patients with EC with either distant metastasis, postoperative recurrence, or unresectable lesions treated using docetaxel plus nedaplatin and 5-FU, 62.79% achieved ORR, with a median OS and TTP of 10.3 and 6.7 months, respectively [15]. Yoshihiro [16] enrolled 14 patients with previously untreated advanced cervical EC with T3-T4 tumors (*n* = 7) and/or M1 staging (*n* = 7). All patients received an infusion of docetaxel and nedaplatin plus oral S-1. The response rate was 78.6% (11/14), including a CR rate of 35.7% (5/14). These results suggest that the efficacy of chemotherapy has achieved a plateau. Thus, new strategies are needed to improve the outcome of stage IV ESCC.

Stage IV EC requires treatment of both the primary tumor and metastatic lesions. Hence, the ideal treatment modality should simultaneously control both, and the addition of targeted radiotherapy to systemic chemotherapy may be particularly effective against the primary tumor. However, at the time this paper was written, limited literature was available on the potential benefits of radiotherapy added to palliative chemotherapy for stage IV ESCC. In some studies, radiotherapy was administered to patients after induction chemotherapy. For instance, Lee et al. [17] treated 74 patients with stage IV EC with two cycles of induction chemotherapy, after which patients classified as M1a and M1b (nonvisceral lymph node metastases) were treated with 54 Gy of radiotherapy concurrently with weekly capecitabine and CDDP. Only three out of 18 M1a patients (16.7%) and four out of 27 M1b patients (14.8%) attained PR after induction chemotherapy. However, the response rates increased to 77.8 and 62.9% after chemoradiation. Thus, the addition of radiotherapy after induction chemotherapy may significantly improve the tumor response in patients with stage IV EC. In another prospective nonrandomized study [18], 47 patients with stage IV ESCC were treated with two to six cycles of induction chemotherapy (PTX plus CDDP). Patients who achieved CR, PR, or SD were non–randomly assigned into the radiotherapy group or nonradiotherapy group, and the radiotherapy group demonstrated longer median OS (13 months versus 11 months) and TTP (10 months versus five months).

While radiotherapy after induction chemotherapy appears promising compared to chemotherapy alone, there are potential disadvantages. For instance, induction chemotherapy delays radiotherapy, and some patients experiencing tumor progression during induction chemotherapy may lose the opportunity for radiotherapy. Thus, in several recent studies, CCRT has been examined for the treatment of stage IV EC.

A retrospective study of 40 metastatic EC cases treated with CCRT (40 Gy of radiation plus combined 5-FU and CDDP) reported an ORR of 55%, a median OS of 10.1 months, and a median PFS of 4.6 months [19]. Another retrospective study of 50 patients with stage IV EC treated with CCRT, among whom 90% received a total radiation dose of at least 50 Gy and a median of four cycles using 5-FU and CDDP, reported a primary tumor ORR of 80%, median PFS of 4.7 months, and OS of 12.3 months [20]. Lee et al. reported a superior one-year OS with CCRT compared to palliative chemotherapy alone (45% versus 18%) in 67 patients with inoperable stage IV EC [21]. There is also a prospective study reported by Ishida K et al. [22], which is concerned with the effect of CCRT for patients with advanced esophageal squamous cell carcinoma. A total of 60 patients with either T4 tumor or distant lymph node metastasis were enrolled. All the patients received CDDP plus 5-FU chemotherapy and a total dose of 60 Gy radiotherapy. The overall response rate and complete response rate were 68.3 and 15%, respectively. The median survival time was 305.5 days, and the 2-year survival rate was 31.5%. Collectively, these studies suggest that CCRT is beneficial for stage IV EC, but a definitive conclusion is currently not possible owing to the small number of patients. However, the current retrospective study of 141 patients reached similar conclusions, as we found that patients with stage IV ESCC treated with CCRT achieved significantly better primary tumor responses (74.5% versus 45.3%, *p* = 0.001), OS (median: 14 months versus 11 months, *p* = 0.007), and PFS (median: eight months versus six months, *p* = 0.005) than with chemotherapy alone. Our univariable and multivariable analyses further revealed that CCRT is an independent prognostic factor for OS. Despite the retrospective design of the study, these results provide additional evidence for the benefits of CCRT in advanced ESCC.

The radiation doses to the primary tumor ranged from 40 Gy to 60 Gy among the studies cited above. However, the optimal radiation dose for stage IV EC is still controversial. The dose of radiotherapy has been found to correlate significantly with tumor responses in patients with EC. In the current study, we administered a higher radiation dose (median: 56.4 Gy) than that applied in a previous retrospective study (40 Gy) [19], and most (74.5%) patients in our study achieved a higher primary tumor response. The reduction in primary tumor volume during radiotherapy may prolong the survival of patients with stage IV EC. Whether an even higher dose would lead to better tumor regression and longer OS is unknown, but it is important to balance palliative outcomes with the cost and greater toxicity of higher irradiance in stage IV ESCC, especially in those patients who cannot expect a cure.

The stage of cancer at diagnosis is generally the most important factor for determining prognosis. Dashan et al. reported that the prognosis of patients with EC with a solitary distant metastasis was markedly superior to that of patients with multiple metastases [5]. Chen et al. also reported that the survival duration of patients with EC with a solitary distant organ metastasis was longer than that of patients with multiple distant organ metastases [23]. Our multivariate analysis also showed that solitary metastasis was a statistically significant predictor of prolonged OS, as patients with multiple metastases exhibited a 1.51-fold greater risk of mortality (95% CI: 1.036–2.212). Hellman and Weichselbaum [24] proposed a state of oligometastases in which metastases are limited, and patients with oligometastases may benefit from a combination of systemic and local therapy [25, 26]. From the available data, we cannot provide an exact definition of oligometastases in EC; thus, further studies are needed.

Hematological toxicities were the main side effects in this study. The incidence of grade 3 or higher leucopenia was significantly greater in the CCRT group than in the chemotherapy-alone group (41.8% versus 24.4%, *p* = 0.040). It was reported that bone marrow hematopoietic precursors within the radiotherapy field demonstrate an acute radiation injury owing to their rapid and constant proliferative state, resulting in myelosuppression [27]. The radiation of the ribs, sternum, or thoracic spine may contribute to the greater myelosuppression observed in CCRT. Alternatively, other treatment-related AEs observed in our study were manageable, including nausea/vomiting, acute treatment-related pneumonitis, and acute treatment-related esophagitis, suggesting that CCRT is generally tolerable and applicable to a substantial proportion of patients with stage IV EC.

The limitations of this study included its retrospective nonrandomized design and single-center data collection. In addition, patients with esophageal bleeding or with complaints of obstruction or dysphagia are more likely to receive concurrent radiotherapy, which introduces a selection bias in the comparison between chemotherapy and CCRT. Nevertheless, the significant improvements in ORR, PFS, and OS observed with CCRT justify randomized prospective clinical trials comparing CCRT to chemotherapy alone for treatment of stage IV ESCC.

## Conclusions

From this nonrandomized, retrospective study, we can conclude that, CCRT is more effective than chemotherapy alone for stage IV ESCC, resulting in better primary responses and survival outcomes with tolerable side effects.

## Abbreviations

AE: Adverse event
CCRT: Concurrent chemoradiotherapy
CDDP: Cisplatin
CI: Confidence interval
CR: Complete response
DCR: Disease control rate
EC: Esophageal cancer
ESCC: Esophageal squamous cell carcinoma
HR: Hazard ratio
IMRT: Intensity-modulated radiation therapy
ORR: Objective response rate
OS: Overall survival
PD: Progressive disease
PFS: Progression-free survival
PR: Partial response
PTX: Paclitaxel
SCC: Squamous cell carcinoma
